# The remote diet intervention to reduce Long COVID symptoms trial (ReDIRECT): protocol for a randomised controlled trial to determine the effectiveness and cost-effectiveness of a remotely delivered supported weight management programme for people with Long COVID and excess weight, with personalised improvement goals

**DOI:** 10.3310/nihropenres.13315.2

**Published:** 2023-08-03

**Authors:** Laura Haag, Janice Richardson, Yvonne Cunningham, Heather Fraser, Naomi Brosnahan, Tracy Ibbotson, Jane Ormerod, Chris White, Emma McIntosh, Kate O'Donnell, Naveed Sattar, Alex McConnachie, Michael E. J. Lean, David N. Blane, Emilie Combet

**Affiliations:** 1 Human Nutrition, School of Medicine, Dentistry & Nursing, University of Glasgow, Glasgow, G31 2ER, UK; 2 School of Cardiovascular and Metabolic Health, University of Glasgow, Glasgow, G12 8TA, UK; 3 General Practice & Primary Care, School of Health and Wellbeing, University of Glasgow, Glasgow, G12 9LX, UK; 4 Health Economics and Health Technology Assessment, School of Health and Wellbeing, University of Glasgow, Glasgow, G12 8RZ, UK; 5 Counterweight Ltd., London, UK; 6 Long COVID Scotland, Edinburgh, UK; 7 Robertson Centre for Biostatistics, University of Glasgow, Glasgow, G12 8QQ, UK

**Keywords:** Long COVID; Randomised Controlled Trial, Weight management, Total diet replacement, Remote delivery, Personalisation, Post COVID-19 Syndrome

## Abstract

**Objectves:**

The Remote Diet Intervention to Reduce Long COVID Symptoms Trial (ReDIRECT) evaluates whether the digitally delivered, evidence-based, cost-effective Counterweight-Plus weight management programme improves symptoms of Long COVID in people with overweight/obesity.

**Methods:**

Baseline randomised, non-blinded design with 240 participants allocated in a 1:1 ratio either to continue usual care or to add the remotely delivered Counterweight-Plus weight management programme, which includes a Counterweight dietitian supported delivery of 12 weeks total diet replacement, food reintroduction, and long-term weight loss maintenance. Randomisation is achieved by accessing a web-based randomisation system incorporated into the study web portal developed by a registered Clinical Trials Unit. We are using an innovative approach to outcome personalisation, with each participant selecting their most dominant Long COVID symptom as their primary outcome assessed at six months. Participants in the control arm enter the weight management programme after six months. We are recruiting participants from social media and existing networks (e.g., Long COVID Scotland groups), through newspaper advertisements and from primary care. Main inclusion criteria: people with Long COVID symptoms persisting > three months, aged 18 years or above, body mass index (BMI) above 27kg/m ^2^ (>25kg/m ^2^ for South Asians). The trial includes a process evaluation (involving qualitative interviews with participants and analysis of data on dose, fidelity and reach of the intervention) and economic evaluation (within-trial and long-term cost-utility analyses).

**Anticipated results:**

The recruitment for this study started in December 2021 and ended in July 2022. Project results are not yet available and will be shared via peer-reviewed publication once the six-months outcomes have been analysed.

**Trial registration:**

Current Controlled Trials ISRCTN12595520.

## Plain language summary

While most people infected with COVID-19 recover within a short amount of time, some people continue to have symptoms for 12 weeks or longer. This condition is known as Long COVID. Roughly two-thirds of people with Long COVID are overweight, a proportion similar to that found in the general population. Being overweight may worsen symptoms such as fatigue, breathlessness and pains. Weight management programmes in adults with overweight/obesity can reduce such symptoms, however we do not know how effective intentional weight loss is to reduce symptoms for people with Long COVID. The aim of this project is to test a well-established weight management programme, delivered and supported remotely, in people with Long COVID.

The trial is conducted with 240 people with Long COVID, identified through their GP, patient groups, social media, or newspaper advertisements. A total of 120 individuals will receive the personalised, professionally supported weight management programme (treatment group), and 120 participants are allocated to usual care (control group). The one-year long weight management programme involves 12 weeks of total diet replacement (TDR) using soups and shakes, followed by food reintroduction and weight maintenance. Food based alternatives are available to those who are unable, or prefer not to, follow the TDR approach. The two groups will be compared for Long COVID symptoms, weight loss, quality of life and value for money after six months. After six months, the weight management programme will also be provided for the control group. Experiences while on the programme will be documented for 12 months for all participants.

People with Long COVID have been involved extensively in developing this project. Their priorities are to reduce symptoms like fatigue, breathlessness and pain. They are keen to explore if effective weight management would help their symptoms and overall functioning, especially a programme that can be followed remotely from home. A group of patients and other stakeholders has been set up to provide advice throughout the project.

## Introduction

After a COVID-19 infection, some people continue to experience long-term effects of the illness known as Long COVID (other names include post-COVID-19 condition, post COVID-19 syndrome, long-haul COVID, and post-acute sequelae of SARS CoV-2 infection (PASC)), a multi-system condition with symptoms which vary from person to person in number, type, severity, and duration. The proportion of people who develop Long COVID remains uncertain – estimates in the UK range from 14% ( Office for National Statistics, 2021) to 38% (REACT-2 Study ( Whitaker *et al.*, 2022)).

Commonly reported symptoms include fatigue, headaches, breathlessness, loss of taste/smell, cognitive dysfunction, and joint and muscle pain ( Davis *et al.*, 2021; Lopez-Leon *et al.*, 2021; Sudre *et al.*, 2021) with over 50 long-term effects of COVID-19 reported ( Lopez-Leon *et al.*, 2021). Long COVID presents a major public health issue, with substantial impacts not only on the health service but also individuals and families affected, with substantial impacts on quality of life and economic costs (loss of work, care, and support costs) ( Aiyegbusi *et al.*, 2021; Michelen *et al.*, 2021; Vaes *et al.*, 2021). At the time of writing, the pathophysiology of Long COVID is still not well understood. Several underlying disease mechanisms may contribute to different Long COVID symptoms. For instance, research has suggested the following potential contributors: persistent viral infection or reservoir of non-infections remnants; lasting tissue damage due to direct viral toxicity; dysbiosis; small (or ‘micro’) blood clots; auto-immunity; and inflammation following initial infection ( Choutka *et al*., 2022; Nalbandian *et al.*, 2021; Phetsouphanh *et al.*, 2022).

Obesity is a risk factor for severe COVID-19 infection, with each additional body mass index (BMI) unit above 23 kg/m ^2^ increasing the risk for a COVID-19 related hospitalisation by 5% (HR 1.05 [95% CI 1.05 – 1.05]) ( Gao *et al.*, 2021). Mendelian randomisation analyses suggest that this link between genetically higher BMI and COVID-19 hospitalisation may be even stronger (OR 1.14 [95% CI 1.07 – 1.21] per additional BMI unit) ( Leong *et al.*, 2021; Sattar & Valabhji, 2021). Whilst Long COVID can happen following an initial infection at any disease severity, there is evidence suggesting an association between Long COVID and severity of the acute infection: in the COVID-19 symptom study, those who had at least five symptoms in the first week of the acute illness had increased odds of experiencing Long COVID symptoms for more than four weeks (OR 3.95 [95% CI 3.10 – 5.04]) ( Sudre *et al.*, 2021).

Being female and/or living with obesity is associated with lesser likelihood of a full COVID-19 recovery at one year (OR 0·68 and 0·50 respectively) ( Evans *et al.*, 2022). In a meta-analysis of 10 UK longitudinal studies, excess weight (BMI > 25 kg/m ^2^) was associated with increased odds of symptoms lasting for at least four weeks (OR = 1.24, CI 1.01 – 1.53) ( Thompson *et al.*, 2022). In non-hospitalised adults (UK-based primary care data), a higher BMI (>30 kg/m ^2^) was associated with a 10% increased risk of symptoms persisting at least 12 weeks in comparison to those with a BMI in the normal range (adjusted HR 1.10 [95% CI 1.07 – 1.14]) ( Subramanian *et al.*, 2022). In a large community-based sample (REACT-2 study, England), the risk of Long COVID increased with increasing adiposity (overweight: OR 1.15 [95% CI 1.09 – 1.21]; obesity: OR 1.39 [95% CI 1.32 – 1.48]) ( Whitaker *et al.*, 2022).

Potential pathways proposed to link overweight/obesity to more adverse COVID-19 illness include cardiovascular and metabolic impairments, reduced lung function, and a chronic low-grade inflammatory state related to obesity and excess ectopic fat, which may amplify the immune response during COVID-19 infection ( Sattar *et al.*, 2020).

In studies conducted prior to the COVID pandemic, weight loss has been shown to lower inflammatory levels ( Marfella *et al.*, 2004; Sheu *et al.*, 2008; Vilarrasa *et al.*, 2007; Welsh *et al.*, 2010), and may improve cardiometabolic health, lung function, and quality of life ( Aucott *et al.*, 2005; Avenell *et al.*, 2004; Stenius-Aarniala *et al.*, 2000). Some of the most common symptoms of Long COVID (fatigue, pains, breathlessness) are also common with overweight/obesity, and helped by weight loss ( Riddle & Stratford, 2013; Stenius-Aarniala *et al.*, 2000) – there is a need to update the evidence-base to document whether weight management can play a role in Long COVID rehabilitation for people living with excess adiposity. With metabolic complications of obesity (type 2 diabetes (T2D), hypertension, dyslipidaemia) shown to be rapidly reversible with weight loss and loss of ectopic fat ( Aucott *et al.*, 2005; Lean *et al.*, 2018), weight management is also relevant in the context of increased risk and associated burden of incident diabetes in Long COVID ( Xie & Al-Aly, 2022).

To mitigate the impact of Long COVID, one of the top research priorities identified by a global public forum is to investigate the impact of non-pharmaceutical interventions and anti-inflammatory therapeutics ( Carson & Long Covid Forum Group, 2021). In alignment with this goal, the purpose of the ReDIRECT study is to investigate whether supported weight loss can help alleviate symptoms of Long COVID in people who are living with excess adiposity. ReDIRECT uses an existing dietary weight management programme, shown to be safe, effective, and cost-effective in achieving and sustaining mean weight loss of >15 kg and remission of T2D (Diabetes Remission Clinical Trial (DiRECT)) ( Lean *et al.*, 2018; Xin *et al.*, 2020).

The ReDIRECT randomised controlled trial, including the diet intervention, is delivered entirely remotely using digital technology. The trial design involves parallel groups allocated in a 1:1 ratio, with a wait-list entry to the weight management intervention for the control group, and a framework to test superiority of the intervention arm. ReDIRECT uses an innovative approach to personalisation, with each participant selecting their dominant symptom as primary outcome. This approach is particularly relevant in the context of the broad spectrum of Long COVID symptoms reported. 

## Study objectives

### Primary research objective

The aim of this research is to implement a remotely delivered digitally supported weight management programme for people with Long COVID, with personalised improvement goals; and to determine the effectiveness and cost-effectiveness of this intervention for improving symptoms.

The primary research objective is to evaluate at six months whether:

1. Effective weight management improves self-selected personalised primary symptoms of Long COVID *vs* standard care (in absence of established treatment for Long COVID).
2. The weight management intervention improves wider health, quality of life and psychological outcomes *vs* standard care.
3. Symptom improvements persist to 12 months.

### Secondary research objectives

1. Assess the implementation of the intervention in terms of dose, fidelity, and reach, and explore differences by sociodemographic characteristics (age, sex, ethnicity, socio-economic status (SES)).
2. Explore how contextual factors influence variations in implementation and effectiveness and identify barriers and facilitators to delivery.
3. Explore the experience of the intervention from the perspective of participants, including acceptability, patterns of use, and barriers and facilitators to use.
4. Evaluate within-trial and long-term cost-effectiveness of the intervention.

## Methods

### Patient and public involvement (PPI)

PPI has been integral to the development of our research. We consulted with people from the Long COVID Scotland support group at an early stage to obtain data on the acceptability and desirability of the ReDIRECT study. A lay co-applicant with lived experience of Long COVID attends Study Management Group (SMG) meetings. We have also established a COVID-19 PPI group, involving people with Long COVID who are independent of the study. The PPI group and lay co-applicant review the ethics application, social media outputs (including the website design), recruitment, analysis and dissemination plans.

### Study design

This registered randomised controlled trial (ISRCTN12595520, protocol registered 25/11/2021) uses a wait-list controlled design, with participants allocated at baseline in a 1:1 ratio to one of two groups using a mixed approach of minimisation (80%) and randomisation (20%). The treatment allocation is performed by a registered Clinical Trials Unit and is designed to maintain balance with respect to the dominant Long COVID symptom (fatigue, breathlessness, pain, anxiety/depression, other), sex (male, female, or other), age (<50, ≥50), self-reported ethnicity using UK Census classifications (White, South Asian, Other) and postcode-based index of multiple deprivation (IMD)) (<median, ≥median).

Participants are randomised to one of two study groups:

- Treatment: evidence-based weight management programme (Counterweight-Plus, delivered and supported by Counterweight Ltd)
- Control: standard care (delayed entry to the Counterweight-Plus programme after six months)

**Figure 1.**
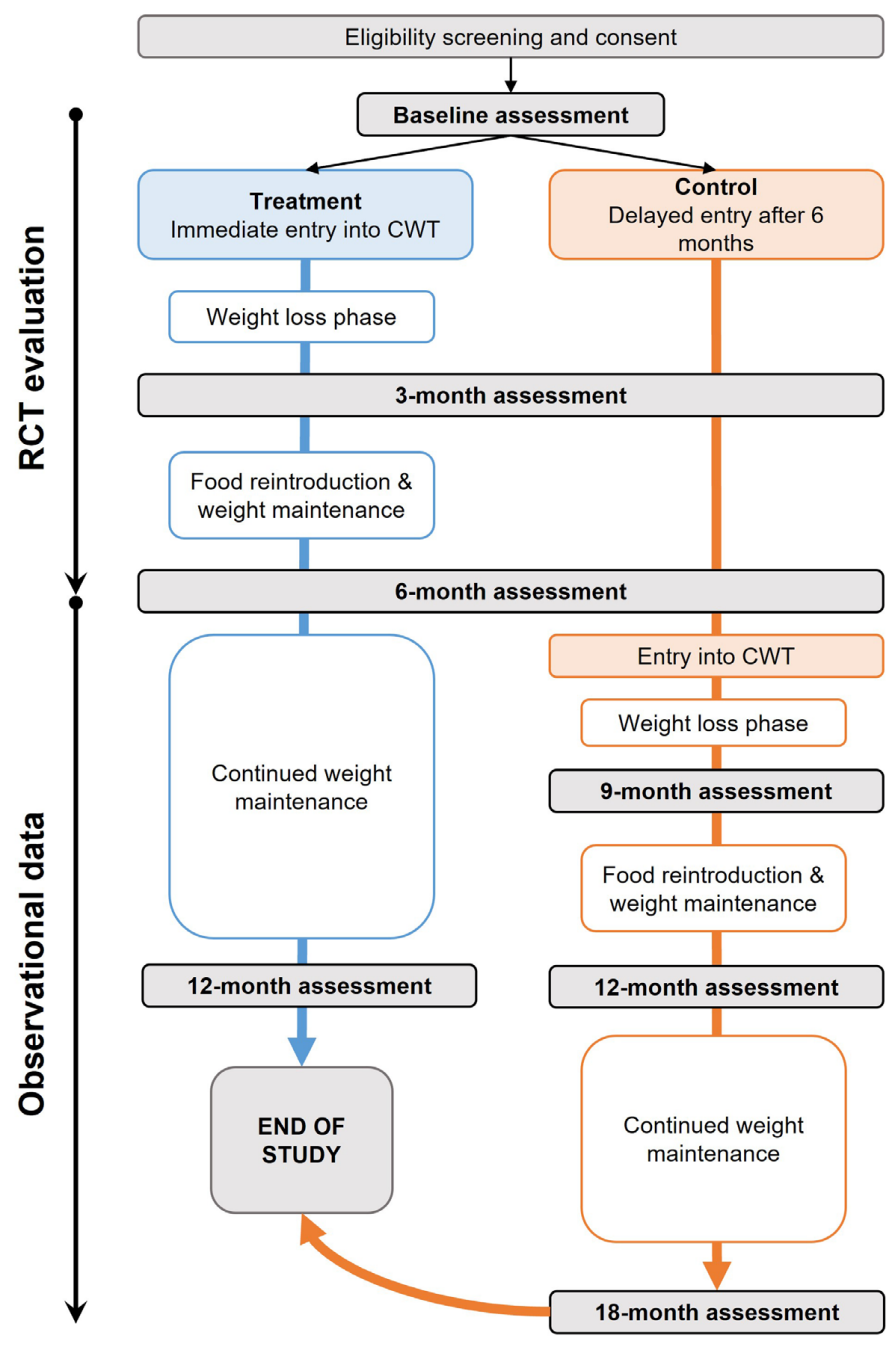
ReDIRECT trial flow diagram. The ReDIRECT Study is a parallel, non-blinded, wait-list controlled study with 240 participants allocated in a 1:1 ratio either to the Counterweight-Plus weight management programme (treatment) or to continue usual care (control group with delayed entry to the Counterweight-Plus programme after 6 months). The effectiveness of the trial will be evaluated after six months. Further data will be collected after six months to the end of the trial for additional observational analyses. Blue: treatment group (immediate entry to Counterweight); Orange: control group (usual care); CWT: Counterweight; RCT: randomised controlled trial.

The weight management programme includes a formula low-energy diet for eight to 12 weeks to induce weight loss, food reintroduction and weight loss maintenance (week 8/12 to month six). The impact of the intervention on the patient’s dominant symptom will be assessed at the six-month mark. After six months, observation will continue up to 12 months for cross-sectional analysis of long-term maintenance of weight loss. Participants in the control group will be offered delayed entry to Counterweight-Plus programme at this point and will add observational data to the 12-month time point, but not form part of the RCT analysis ( Figure 1).

The study is open-label due to the nature of the intervention and participants, researchers (who enrol the participants and organise all follow-ups), and dietitians (who deliver the intervention) are aware of the treatment allocation. However, the study statistician will remain blinded to the allocation until completion of the statistical analysis plan and locking of the database up to six months post-randomisation.

There are no pre-specified criteria for discontinuing the intervention or modifying the allocation. The intervention may be discontinued as per a participant’s wishes, or as a result of adverse events making pursuing the intervention incompatible with care. In the event of a participant discontinuing the intervention, participant follow-up remains in place to collect outcome data.

### Setting

All aspects of the study are delivered fully remotely, including recruitment, giving consent, randomisation, the intervention, and outcome assessments to minimise participant burden and cost. Glasgow is the sole study site, whilst participants are recruited across Scotland, England, Wales, and Northern Ireland.

### Study population and recruitment

We aim to recruit a total of 240 people aged 18 years and above, with self-reported symptoms of Long COVID and overweight/obesity (n = 120 per study arm) from general practice, social media, newspaper advertisements and existing networks, such as national Long COVID groups. Inclusion and exclusion criteria are reported in Table 1. A positive COVID-19 diagnosis is not a requirement for this study due to the unavailability of COVID-19 tests at the start of the pandemic. This inclusive approach was chosen based on feedback from the PPI group and a strong steer from the funder. Reasons for exclusions are fully recorded. There is no exclusion on concomitant care, except when related to the exclusion criteria listed in Table 1.

**Table 1.** ReDIRECT inclusion and exclusion criteria.

Inclusion criteria	People who are aged **≥**18 years
	People with Long COVID symptoms persisting >3 months before first recruitment contact, not currently hospitalised
	People with a body mass index (BMI) ≥27 kg/m ^2^ (≥25 kg/m ^2^ for South Asians).
Exclusion criteria	People who have had lengthy hospitalisations (>10 days) or intensive care unit admissions related to COVID-19
	People who are currently on insulin or anti-obesity drugs
	People who have had a proven myocardial infarction within the last 6 months
	People with severe mental illness, including severe depression or eating disorder
	Women who are pregnant or considering pregnancy
	People who have a history of substance abuse
	People with an active illness likely to cause a change in weight
	People who underwent bariatric surgery within the last 3 years or are planning bariatric surgery
	People with advanced kidney problems (eGFR < 50 ml/min/1.73m ^2^), gallstones or pancreatitis
	People currently participating in another clinical research trial likely to affect diet or weight change
	People with learning disabilities
	People who are unable to understand English (written or verbal)

Recruitment from general practice is facilitated by the NRS Primary Care Network using electronic records search, with potential participants sent an invitation to participate from their GP on behalf of ReDIRECT, including a participant information sheet explaining the study.

The social media strategy includes regular posts and activity on Twitter and Facebook on behalf of the ReDIRECT Study. Prospective participants are to be directed to the study website and to contact the research team via email. In addition, existing groups and organisations have been identified to support dissemination within their networks. We are working with community stakeholders to increase representation of minorities / usually under-represented groups.

Posters have been placed in primary care waiting rooms to remind patients and encourage those interested to respond if they had received an invitation. Patients who respond as interested in participating are contacted by a member of the research team and invited to attend for a remote screening appointment where the study is fully explained and discussed. Informed consent is secured by the study researchers from all participants, as per trial protocol and for optional indefinite long-term data collection from their medical records and through national records linkages. Participant information sheets and consent forms are sent and returned by emails, initialled and signed (documents available on the ReDIRECT study website https://redirectstudy.co.uk/publications/).

Recruitment began in December 2021 and was completed in July 2022.

### Weight management programme

The format and support of the intervention is delivered remotely by the evidence-based Counterweight-Plus Programme ( Lean *et al.*, 2018). All support is delivered by registered dietitians. Counterweight-Plus is a structured intervention utilising total diet replacement (TDR), and behaviour change to help people lose at least 15 kg (or 15% of their body weight if over 100 kg) and keep it off long term. Recognising that fatigue and mobility difficulties are frequent among people living with Long COVID and among those living with obesity, the programme is delivered to participants’ homes and supported entirely remotely and patient-reported outcomes are used to maximise acceptability and protocol adherence.

The programme runs for 12 months and is divided into two phases:

### Weight loss induction phase (from week 0 to week 8–12)

Counterweight provides 8–12 weeks of a low-energy, nutritionally complete, formula diet (850 kcal/day) to replace all meals (total diet replacement, TDR), aiming for ≥15 kg (or 15% of their body weight if over 100kg) weight loss. Products include a variety of soups and shakes provided free of cost to the participant. Those who are unable, or prefer not to, follow the TDR formula diet (10% in the published DiRECT study ( Lean *et al.*, 2018)) are offered an alternative approach providing 1200 kcal/day, either a meal replacement diet in which a maximum of two meal replacements are consumed per day or a food-based low-carb or low-fat approach. Food used to supplement the TDR or for fully food-based plans is purchased by the participants and not provided by the study. To increase adherence to the programme, support is tailored to individual participants, allowing for flexibility around commitments or life events. Further strategies are pre-emptively discussed, e.g., the possibility of adding in one meal/day or including ‘days off’, with encouragement to resume the full TDR at subsequent visits and to maintain whatever weight has already been lost and moving onto the maintenance phase early if necessary to avoid dropping out altogether.

### Weight loss maintenance phase (week 8/12 – six months)

Food is reintroduced stepwise while reducing TDR. The transition period to a full food-based diet lasts between 4–12 weeks (usually eight weeks) and is individualised to participants. A management plan will be provided along with support for standard evidence-based behaviour change techniques, including self-monitoring of behaviours and behavioural outcomes, goal-setting and self-rewards, action planning, and problem solving. During this time, participants measure their weight weekly and enter this into the Counterweight App to monitor progress with weight loss maintenance.

Relapse treatments are available for >2 kg weight gain. These include reinforcing behaviour change techniques and the option to return to TDR, a 1,200-kcal meal replacement diet, or a 1,200-kcal low carb or low-fat diet for two weeks. Alternative evidence-based dietary strategies are considered on an individual basis (intermittent or alternate-day fasting, time-restricted eating).

Counterweight dietitians provide personalised support throughout the 12-month intervention via text chat, video or telephone, in-app weekly monitoring, nudges, and personalised messaging. Remote appointments are offered on a monthly basis, following initiation into the programme, however, these appointments are flexible to the needs of individuals.

Group support is facilitated using group text chat, and a Facebook community for all Counterweight users.

### Medication review

Each participant is asked to report any prescribed medications at baseline. The Counterweight team follows the published medication withdrawal and reintroduction protocol as used in the DiRECT study. Weight loss is likely to result in a reduction of blood glucose levels and blood pressure. As a safety measure to avoid hypoglycaemia and postural hypotension, it is recommended that all anti-diabetic, anti-hypertensive, and diuretic medication should be stopped on the day TDR is commenced. Blood glucose levels and blood pressure are monitored at each review appointment and, if necessary, medication is reintroduced following a defined protocol. Any such actions, as well as observations and intervention results, which may pose a risk to the participant’s health are discussed between Counterweight dietitians, the participant, and the participant’s general practitioners (GPs). In addition, study clinicians are on hand to provide supplementary advice and guidance regarding participant medication where required.

### Study outcome measures

Outcomes are all measured for both groups at baseline, three, and six months. Follow-ups for cross-sectional analysis are measured at 12 months in the treatment group and nine, 12, and 18 months for the control group, with the exception of height, which is recorded at baseline only.

Due to geographical constraints for this UK-wide trial coupled with COVID-19 constraints, all trial outcomes are self-reported, using a bespoke electronic case report form (eCRF). Remote measurement is anticipated to minimise participant burden and cost and to maximise retention. To facilitate self-reported measurements of weight and blood pressure, participants receive digital scales (Model UC-502, A&D Instruments Ltd, Abingdon, UK) and fully automatic blood pressure monitors (arm type monitor TMB-1970, Kinetik Medical Devices Ltd, Redhill, UK) by post. Other measurements are collected through the eCRF / web-based questionnaires, with all entries verified by the study researchers, who are available to liaise and assist participants throughout the study.

### Primary and secondary outcome measures

All participants are asked to fill in online questionnaires related to a range of identified Long COVID outcomes ( Table 2). The primary outcome is a continuous measure derived from the symptom score for the self-selected dominant Long COVID symptom, nominated by each participant at baseline.

**Table 2.** ReDIRECT trial outcome measures.

	Primary outcome measures	Scales / Tools	References
Self-selected Long COVID symptom	Fatigue	Validated Chalder Fatigue Scale CFQ-11	( Chalder *et al.,* 1993)
	Breathlessness	Modified MRC Dyspnoea Scale	( Bestall *et al.,* 1999)
	Pain	P4 Numeric Pain Rating Scale	( Spadoni *et al.,* 2003)
	Anxiety and depression	Hospital Anxiety and Depression Scale (HADS) questionnaire	( Zigmond & Snaith, 1983)
	Other	Visual Analogue Scale (0-10) for other symptoms with no pre-specified scale	
	Secondary outcome measures	Scales / Tools	References
All non-selected primary Long COVID symptoms	Fatigue, breathlessness, pain, anxiety/depression, other	As above	As above
Body composition and health	Weight	Digital scales (Model UC-502, A&D Instruments Ltd, Abingdon, UK)	
	Height		
	Blood pressure	Arm type monitor (TMB-1970, Kinetik Medical Devices Ltd, Redhill, UK)	
	COVID vaccination		
	Medication		
Other	Quality of life	EQ-5D 5L	( Buchholz *et al.,* 2018)
	Work productivity	Work Productivity and Activity Impairment	( Reilly *et al.,* 1993)
	Healthcare utilisation	Bespoke questionnaire	
	Food expenditure	Bespoke questionnaire	

Secondary outcome measures include all non-selected primary Long COVID symptom outcomes (fatigue, breathlessness, pain, anxiety/depression, other), as well as weight and blood pressure (both self-measured using equipment provided and self-reported via the online questionnaire at assessment timepoints), and other health and wellbeing outcomes outlined in Table 2.

### Intervention adherence monitoring

Adherence to the weight management intervention is monitored using a combination of objective measurements via number of diet products sent to the participant over time, via dietetic consultations, and via engagement with the digital resources made available. These quantitative data will be used in the process evaluation to describe factors such as fidelity to the intervention, dose (based on interactions with the programme) and reach and to examine mediators and moderators of effects.

### Safety reporting

Adverse events (AEs) will not be reported during the course of the trial. Counterweight dietitians will be asked to collate and report only serious adverse events (SAEs) to the study researchers, who will log them into the eCRF and escalate to sponsor pharmacovigilance where related and unexpected.

There is little risk to participants taking part in this study; however, there may be side effects (expected events) from rapid weight loss, relating to the induction period using the total diet replacement sachets (shakes and soups) such as constipation, increased sensitivity to cold, headache, and dizziness (which were commonly reported side effects in the DiRECT study and dissipated over time). Other side effects that are less commonly reported are: fatigue, mood changes, nausea, diarrhoea, indigestion, hair loss. All participants are provided with information on all possible side effects and management strategies should they be problematic. These events would be considered expected within this study.

We do not plan provisions for ancillary or post-trial care. The sponsor (NHS Greater Glasgow and Clyde) will be liable for negligent harm caused by the design of the study. NHS indemnity is provided under the Clinical Negligence and Other Risks Indemnity Scheme (CNORIS).

### Data management

An electronic case report form (eCRF), developed by the Robertson Centre for Biostatistics, captures all data required to meet this protocol’s requirements. Access to the eCRF is restricted, via a trial-specific web portal, and only authorised personnel will be able to make entries or amendments to the participants’ data via the web portal. It is the investigator's responsibility to ensure completion and confirm that the data is accurate, complete and verifiable. Data will be stored in a MS SQL Server database.

Where it is practical, data will be validated at the point of entry into the eCRF. Any additional data discrepancies will be flagged to the investigator and any data changes will be recorded in order to maintain a complete audit trail (reason for change, date change made, who made the change).

The Robertson Centre for Biostatistics systems are fully validated in accordance with industry and regulatory standards and incorporate controlled access security. High volume servers are firewall protected and preventative system maintenance policies are in place to ensure no loss of service or data. Web servers are secured by digital certificates. Data integrity is assured by strictly controlled procedures, including secure data transfer procedures. Data are backed up on-site nightly and off-site to a commercial data vault weekly. The Robertson Centre for Biostatistics has an ISO 9001:2008 quality management system and ISO 27001:2013 for Information Security and is regularly inspected against the standards by the British Standards Institution.

Personal information will be collected via the eCRF to enable participant completion of questionnaires and to facilitate intervention delivery and record linkage. These data items will be encrypted and only those individuals who require to see these data, i.e., research staff, dietitians or the person performing record linkage, will be able to view them. All electronic data will be held securely in accordance with ISO 27001:2013 at the Robertson Centre for Biostatistics, part of the Glasgow Clinical Trials Unit. All Centre staff are required to sign confidentiality agreements and to follow Standard Operating Procedures in accordance with Good Clinical Practice and ISO certification.

The trial data managers, statisticians, health economists or any other staff who will perform data related tasks will only be able to access depersonalised data where the participant’s identifying information is replaced by a unique study identifier. Only those that have been trained and approved will be able to enter or view any data via the web portal.

Participant consent forms will be stored in a secure password-protected folder on the University of Glasgow servers.

Study investigators based at the University of Glasgow and NHS Greater Glasgow and Clyde will have access to the final dataset. This will be restricted to the relevant staff involved in drafting and reviewing the final study report. The final dataset will be backed-up on a secure university folder, with password protection.

### Statistical analysis

At the design stage for the trial, the primary outcome was proposed as the self-selected, most troublesome symptom (fatigue, breathlessness, pain, anxiety/depression, or another symptom as selected by the participant). Since each outcome measure will have a different distribution, the proposal was to standardise each measure by subtracting the mean and dividing by the standard deviation (SD) from those participants who selected that symptom as their most troublesome. The suitability of this measure for the primary analysis will be assessed during the trial, as data accrue, in blinded analyses. Reports to the Trial Steering Committee (TSC) include assessments of the distribution of this measure, and the committee will advise and approve the final choice of primary outcome and its analysis. The TSC will approve the final Statistical Analysis Plan, prior to locking the six-months dataset and unblinding the statistical team.

The proposed analysis of the primary outcome at six months will be to compare randomised groups using a linear regression model, adjusting for the baseline value of the primary outcome, and factors used to balance the randomisation/minimisation procedure. We do not plan interim analyses. The intervention effect will be reported as the adjusted mean difference between groups, with a 95% confidence interval, and p-value. The analysis will be according to the intention-to-treat principle, in that participants will be analysed according to their randomised group, regardless of compliance with the intervention.

Secondary analyses will use linear or logistic regression, as appropriate, to estimate between-group differences or odds ratios for secondary outcomes. Missing data will not be imputed initially, but the sensitivity of main analyses will be explored using multiple imputation, and analyses weighted by the inverse probability of follow-up, based on a non-parsimonious model of the probability of being followed up, as a function of baseline characteristics. Associations between baseline characteristics, primary and secondary outcomes will be explored by extending the main analysis regression models.

Subgroup analyses will be carried out by including the subgroup variable and its interaction with treatment group in these models. Prespecified subgroups of interest will include sex, age, ethnicity, socioeconomic status (based on postcode index of multiple deprivation), main symptom, and baseline BMI. We will also consider analysis by recruitment source (social media vs primary care) if possible. Additional exploratory analyses will investigate if short-term differences between randomised groups (particularly early weight loss) are predictive of six-month intervention effects. Additional observational analyses will explore within-group changes in outcomes between six and 12 months in both randomised groups, and predictors of long-term outcomes in the intervention arm of the trial. 

### Sample size

Assuming the SD of the primary outcome at follow-up (derived from the symptom score at follow-up, standardised using the same mean and SD as used at baseline) is one, then to have 90% power to detect a between group difference of 0.5 at follow-up, at a 5% significance level, requires a sample size of 86 per group with follow-up data. To allow for attrition, we aim to randomise 240 people.

If the intervention proves to be acceptable and safe, the 120 participants originally allocated to the control group will be offered the intervention as a delayed entry group. Their results (some may exceed the lifetime of the project) will be analysed as observational data and used to increase the study power for hypothesis generation, e.g., to explore relationships between extent of weight change and Long COVID symptoms.

### Process evaluation

A mixed-methods process evaluation will be conducted. We are collecting quantitative data (such as intervention dose, adherence to the intervention, and reach) and qualitative data to understand barriers and facilitators to the implementation of the intervention, how the intervention was experienced, and to explore unanticipated impacts of the intervention.

Qualitative data are collected through longitudinal semi-structured telephone or video interviews at baseline (n=30), six (n=15), and 12 months (n=15). The reason for the reduced number of interviews at 6 and 12 months is partly to account for drop out, but is mainly pragmatic, taking account of a) participant burden (giving additional time to interviews for a group of participants with high prevalence of fatigue and ‘brain fog’) and b) researcher time and resource to undertake and analyse a large number of interviews. Interviews will focus on patient expectations, motivations, experience of the intervention, impact on Long COVID symptoms, and contextual factors that affect (and may be affected by) implementation, intervention mechanisms and outcomes. Focus group interviews are also held with dietitians to explore their understanding of Long COVID and their experiences of supporting people with Long COVID with the ReDIRECT weight management intervention. Quantitative and qualitative data will first be analysed separately and then considered together, to further our understanding of participating in ReDIRECT.

Interviews and focus groups will be transcribed and analysed inductively first to identify broad themes and concepts in the data. Data will then be mapped to the constructs of Normalisation Process Theory (NPT) to understand the ‘work’ that participants do to incorporate the intervention into their daily routine ( May *et al.*, 2009). Coding and mapping to NPT will be a reflexive process involving several members of the team; discrepancies in coding and data interpretation will be discussed within the team and also with our PPI reps to ensure that the voice of those living with Long COVID is reflected in our analyses.

### Economic analysis

Building directly on the methods employed in the published within-trial economic evaluation of the Counterweight-Plus intervention in the DiRECT trial ( Xin *et al.*, 2020), the economic analysis will be carried out according to a detailed Health Economic Analysis Plan (HEAP). The HEAP will detail the methods to be employed for the within trial economic evaluation (and subsequent long-term cost-utility model). Intervention costs will be re-estimated from the original DiRECT trial (given online delivery) and all resource use will be measured and valued. The EQ-5D-5L preference-based quality of life instrument will be combined with trial duration to estimate Quality Adjusted Life Years (QALYs). Differences in cost and QALYs between the intervention and control groups will be estimated using survival analysis and presented as an Incremental Cost-Effectiveness Ratio (ICER) ( Williams *et al.*, 2017).

In line with NICE guidance, the primary within-trial and lifetime cost-utility analyses will adopt an NHS and personal social services (PSS) perspective ( National Institute for Health and Care Excellence, 2013). Secondary analyses, with specific PPI input, will consider a broader societal perspective (personal out-of-pocket expenses, productivity and food and drink expenditure). Work-related productivity, healthcare resource utilisation and other relevant personal costs (including weekly food, drink and lifestyle expenditure and over the counter medications) will form the basis of a bespoke resource use questionnaire. This health economic questionnaire will be adapted to the participant population, tested with the PPI group, and administered at baseline, three, six, and 12 months ( Combet *et al.*, 2022a).

Within-trial costs and QALYs will be adjusted for baseline covariates. A probabilistic sensitivity analysis will be conducted, with the resultant ICERs presented on a cost-effectiveness plane and used to construct a cost-effectiveness acceptability curve (CEAC). A discount rate of 3.5% will be used and if cost/outcome differences transpire, a longer-term cost-utility model will be developed, building on the existing DiRECT model. All analysis and reporting guidelines will be adhered to, according to the updated Consolidated Health Economic Evaluation Reporting Standards (CHEERS) checklist ( Husereau *et al.*, 2022).

### Funding, ethical approval, and role of study sponsor and study funder

NHS Greater Glasgow and Clyde (NHSGGC) is the sponsor of the trial. The study is funded through an award from the National Institute for Health and Care Research (NIHR) to the University of Glasgow following peer review with the award reference number COV-LT2-0059. Counterweight Ltd, sub-contracted to the University of Glasgow, is the study intervention provider.

Ethical approval was obtained from the South-East Scotland Research Ethics Committee 01 (reference number: 21/SS/0077). At the time of writing, the latest version of the protocol was v1.3, dated June 15 ^th^, 2022.

Study sponsor and funder play no role and have no authority in / over study design; collection, management, analysis, nor interpretation of data; writing and submission of study findings for publication.

NHSGGC conducts monitoring and/or audit of studies on a risk basis and in accordance with local Standard Operating Procedures.

### Trial management

The study is coordinated by the Study Management Group (SMG), consisting of all co-applicants, the project manager and representatives from both NHSGGC and other organisations relevant to the study. The role of the group is to monitor all aspects of the conduct and progress of the study, ensure that the protocol is adhered to and take appropriate action to safeguard participants and the quality of the trial itself. The SMG meets on a bi-monthly to monthly basis.

A Trial Steering Committee (TSC) provides overall supervision of the trial and ensures that it is being conducted in accordance with the principles of good clinical practice (GCP) and the relevant regulations. It includes an independent chairperson, an independent statistician and clinical trials specialist, an independent health economist, the sponsor and an independent patient representative. The TSC meets every four months and reviews the trial protocol, agrees substantial protocol amendments, and provides advice to the investigators on all aspects of the trial. Decisions about continuation or termination of the trial or substantial amendments to the protocol are the responsibility of the TSC who will advise the sponsor.

As this study is not classed as a Clinical Trial of an Investigational Medicinal Product (CTIMP), there is no Data Monitoring Committee (DMC) for ReDIRECT.

### Dissemination policy

NHSGGC and the University of Glasgow are joint controllers of the data arising from this study. Once the study has been completed, a final report will be prepared for publishing purposes, and to feedback research results to both sponsor and the ethics committee (REC). This will be provided to sponsor/REC via email and will be made accessible to the wider research community on international study registry websites such as clinicaltrials.gov or EudraCT. There are no sponsor review requirements on publications. Reporting of this protocol has followed the Standard Protocol Items: Recommendations for Interventional Trials (SPIRIT) guidelines ( Chan *et al.*, 2013) ( https://redirectstudy.co.uk/publications/) and trial results will be reported following the Consolidated Standards of Reporting Trials (CONSORT) guidelines ( Combet *et al.*, 2022b).

The main study documentation – study protocol and full study report – will be made accessible to the wider research community on international study registry websites such as clinicaltrials.gov or EudraCT within one year of study opening. The dataset will be made publicly available in a curated, de-identified (in accordance with the Safe Harbor method) and minimised state to mitigate ethical concerns around participants identification and data processing.

Participants will have the opportunity to request their results if they so wish. Participants will be advised upon requesting results that these will be made available once data analysis has been completed and/or the final study report has been compiled.

Study news and findings are communicated to the public via newsletters, the website, funder networking events, a stakeholder meeting, and otherwise opportunity-dependent channels.

Key contributors to the protocol and final report will be noted as authors, as will study researchers who contribute significantly to the running/management of the study. All will be provided with a draft copy of the final report for review prior to publication. Criteria for individually named authors or group authorship will adhere to that of The International Committee of Medical Journal Editors.

## Discussion

The aim of the ReDIRECT study is to test the effectiveness and cost-effectiveness of an evidence-based weight loss programme for people with Long COVID and overweight/obesity. Long COVID is a term used to describe the lasting impacts of a COVID-19 infection which manifest in a broad range of symptoms varying widely in severity and duration. In the UK, the Office for National Statistics (ONS) report in August 2022 estimates that 1.4 million people reported ongoing symptoms for more than 12 weeks and more than 750000 people reported that symptoms had persisted for at least one year ( Office for National Statistics, 2022). People of all ages were affected, including children and adolescents, and many people in the survey reported adverse effects on their daily life and work. Of note, being female was associated with higher odds (OR ~1.5) of developing Long COVID ( Subramanian *et al.*, 2022; Thompson *et al.*, 2022; Whitaker *et al.*, 2022). In addition, in the post-hospitalisation COVID-19 (PHOSP-COVID) study, those who were female and/or living with obesity were less likely to report full recovery at one year (OR 0·68 and 0·50 respectively) ( Evans *et al.*, 2022). Based on these findings, we anticipate recruitment of a higher proportion of female participants.

According to the ONS reports, since April 2021, the estimated number of self-reported Long COVID patients has increased steadily every month. The long-lasting duration and in some cases debilitating and life-changing severity of symptoms, as well as additional complications suggested by emerging data on increased risk and burden of incident diabetes in Long COVID ( Xie & Al-Aly, 2022), will be a major public health concern over the coming years. Currently, there is no specific treatment for Long COVID available, and care involves the individual management of symptoms.

The PHOSP-COVID study noted that obesity was associated with non-recovery and severity of Long COVID, warranting research on whether weight reduction (using either pharmacological and non-pharmacological approaches, alone or in combination) could be part of Long COVID rehabilitation strategies ( Evans *et al.*, 2022). In this study, we aim to establish the impact of intentional weight loss on Long COVID symptoms in people living with excess adiposity. There are no current nutritional recommendations for patients with Long COVID, with uncertainty surrounding the suitability of physical activity and dietary approaches in Long COVID ( Barrea *et al.*, 2022). Weight management may benefit Long COVID patients with overweight or obesity in several ways. In the context of obesity and overweight, weight reduction has well described cardiovascular and renal effects ( Cohen & Cohen, 2015). Weight reduction also improves inflammation ( Forsythe *et al.*, 2008). Weight loss has also been shown to improve quality of life, more so in those living with greater excess weight, and to reduce symptoms common to both being overweight and Long COVID, such as fatigue, breathlessness, and pain ( Babb *et al.*, 2011; Buckell *et al.*, 2021; De Lorenzo *et al.*, 1999; Malfliet *et al.*, 2021; Rigamonti *et al.*, 2020).

The weight management programme adapted in this research has proven health benefits in the general population and in world-leading research on remission of T2D – a co-morbidity of obesity and the second most common co-morbidity of COVID-19 (after hypertension) ( Singh *et al.*, 2020). The programme, Counterweight-Plus, has already been subjected to both feasibility and full-scale randomised controlled trials, and was successfully adopted for the Diabetes Remission Clinical Trial (DiRECT) ( Lean *et al.*, 2018).

The dominant symptom of Long COVID is fatigue ( Lopez-Leon *et al.*, 2021), which is often an obstacle to engagement with conventional dietary programmes for weight management. Therefore, interventions must suit people whose symptoms impair mobility and ability to attend external appointments. The study design takes potential barriers into consideration in the following ways. First, the Counterweight-Plus weight management programme is known to be effective, safe, nutritionally complete, and is provided by a recognised NHS provider. Second, the entire programme has been adapted for entirely remote delivery (app, online portal, and personalised professional support via text chat, video, or telephone), extending reach, scalability, and removing the burden of out-of-home study visits. Third, the intervention focusses on diet only with no specific requirement for exercise (which may not be appropriate or acceptable for people living with Long COVID) but an individually tailored guidance on being active. Fourth, the diet intervention simplifies meal planning and decision-making, which is particularly helpful for those experiencing difficulties concentrating or fatigue.

An important feature of the diet intervention is flexibility in the diet plan (within pre-defined ranges) to accommodate individual circumstances and maximize weight loss for the individual. This includes the possibility to end TDR early, if a participant finds it difficult to adhere to, and a variety of other weight loss plans available to participants after discussion with their dietitian. The flexible approach also includes alternative meals in case TDR is not tolerated and relapse-management plans to treat weight regain. A novel statistical design aspect of this trial is the use of a personalised primary outcome to accommodate varying manifestations of Long COVID from person to person – with participants choosing the dominant symptom they would like to see most improved throughout the trial. This is particularly relevant in the context of the multi-system nature of Long COVID as a disease, and the breadth of Long COVID symptoms documented to date ( Lopez-Leon *et al.*, 2021).

## Conclusion

Long COVID is a relatively new condition that remains poorly defined with unclear mechanisms and no direct treatments. The ReDIRECT study investigates whether an evidence-based and professionally supported weight management programme could be an effective non-pharmaceutical strategy to manage symptoms of Long COVID in those living with excess adiposity. The diet intervention is tailored to Long COVID patients in its remote delivery and flexible approach. The study aims to alleviate Long COVID symptoms, improve quality of life, and evaluate the long-term costs (savings) and effects of the intervention on symptoms after six months, with further cross-sectional analysis after 12 months.

## Data Availability

No data are associated with this article. Figshare: ReDIRECT study eCRF questionnaires screenshots. https://doi.org/10.6084/m9.figshare.21270837.v4. ( Combet *et al.*, 2022a). This project contains the following extended data: merged_all_2022-11-08.pdf (Blank English questionnaire developed for the study outlined in this protocol) Figshare: SPIRIT checklist for ‘The remote diet intervention to reduce Long COVID symptoms trial (ReDIRECT): protocol for a randomised controlled trial to determine the effectiveness and cost-effectiveness of a remotely delivered supported weight management programme for people with Long COVID and excess weight, with personalised improvement goals’. https://doi.org/10.6084/m9.figshare.21404901.v1]. ( Combet *et al.*, 2022b). Data are available under the terms of the Creative Commons Zero "No rights reserved" data waiver (CC0 1.0 Public domain dedication).
