# Supplementary material for: The remote diet intervention to reduce Long COVID symptoms trial (ReDIRECT): protocol for a randomised controlled trial to determine the effectiveness and cost-effectiveness of a remotely delivered supported weight management programme for people with Long COVID and excess weight, with personalised improvement goals
Source: NIHR Open Res. 2023 Aug 3;2:57. Originally published 2022 Nov 24. [Version 2] doi: 10.3310/nihropenres.13315.2 (PMC10593354; doi:10.3310/nihropenres.13315.2)
Supplement: Peer review history

# Reviewer report: Reviewer response for version 2

**DOI:** 10.3310/nihropenres.14610.r30135

**Published:** 2023-08-10

**Authors:** Guri Rørtveit

**Affiliations:** 1 Department of Global Public Health and Primary Care, University of Bergen, Bergen, Norway

The paper with edits is now suitable for indexing. The reasons why they designed the study without a positive Covid test as an inclusion criterion are well described. However, possible implications of this decision is needed in upcoming papers presenting results from the study.

Is the study design appropriate for the research question?

Yes

Is the rationale for, and objectives of, the study clearly described?

Yes

Are sufficient details of the methods provided to allow replication by others?

Partly

Are the datasets clearly presented in a useable and accessible format?

Not applicable

Reviewer Expertise:

Primary care, infectious disease, post-infectious symptoms

I confirm that I have read this submission and believe that I have an appropriate level of expertise to confirm that it is of an acceptable scientific standard.

# Reviewer report: Reviewer response for version 1

**DOI:** 10.3310/nihropenres.14440.r28914

**Published:** 2023-02-14

**Authors:** Guri Rørtveit

**Affiliations:** 1 Department of Global Public Health and Primary Care, University of Bergen, Bergen, Norway

The study protocol describes a randomized controlled study (open label) of weight reduction as an intervention to treat Long COVID. Overweight and obese people with Long COVID are eligible for the study, which has already included the number of participants required.

 The theoretic foundation for the study is well presented. The study has had the advantage of strong patient involvement. One result is that the primary outcome is defined by each participant, selecting their most important symptom as the primary outcome. This seems like an innovative and important strategy for this field, where patients experience so many different symptoms post-COVID. It also make the study results particularly relevant for the primary care setting.

 I have a few comments to the protocol:

 First of all, the protocol defines Long COVID as prolonged symptoms, without securing the diagnosis of COVID. The symptoms in question have a high prevalence in the population also before COVID, meaning that it is difficult to decide whether the problem addressed is Long COVID or not related to this specific infection.

 Secondly, the recruitment strategy involves social media as well as primary care. It might be interesting to stratify analyses to investigate if results differ between recruitment areas.

 Thirdly, it wasn’t quite clear to me whether some of the measurements made by the patients (blood pressure, weight etc) were registered digitally directly to the researcher group, or reported by the participant after measurement. Also, how adherence to weight management is assessed was not clearly described.

 The logistics around information security for the data obtained seem well taken care of.

 I suggest referring to Choutka *et al.,* (Nature Medicine 2022) ^1^ regarding possible mechanisms for post-infectious symptoms.

Is the study design appropriate for the research question?

Yes

Is the rationale for, and objectives of, the study clearly described?

Yes

Are sufficient details of the methods provided to allow replication by others?

Partly

Are the datasets clearly presented in a useable and accessible format?

Not applicable

Reviewer Expertise:

Primary care, infectious disease, post-infectious symptoms

I confirm that I have read this submission and believe that I have an appropriate level of expertise to confirm that it is of an acceptable scientific standard, however I have significant reservations, as outlined above.

## References

[1] : Unexplained post-acute infection syndromes. *Nat Med* .2022;28(5) : 10.1038/s41591-022-01810-6 911-923 10.1038/s41591-022-01810-6 35585196

# Reviewer report: Reviewer response for version 1

**DOI:** 10.3310/nihropenres.14440.r28949

**Published:** 2023-02-13

**Authors:** Louisa Ells

**Affiliations:** 1 Obesity Institute, School of Health, Leeds Beckett University, Leeds, UK

This protocol describes a new RCT to determine the clinical and cost effectiveness of a remotely delivered weight management programme for people living with Long COVID and excess weight.

 The Abstract is clear, but the plain English summary does lack detail about what the weight management programme is about i.e. total meal replacement, food reintroduction and weight maintenance support.

 The introduction provides a clear and appropriate background and justification to the study and the research aim and objectives are clear.

 The Methods stated were clear, with appropriate incorporation of PPIE. The study flow diagram was particularly helpful. My only query: if patients opt for the real food option, is the food provided for them, and for those on the soups and shakes presumably this is purchased for the patient - some cost clarification would be useful? In the study outcome measures can you make it clear that baseline assessments are measured for both groups (as I assume this was the case) but the current text isn’t clear. In the process evaluation it is unclear as to why the longitudinal interview numbers reduce over time – is this expected drop out – if so please state what this is based on, if not this could potentially introduce bias.

 The dissemination section seems to be missing any reference to public and patient communication of the study findings.

 The discussion was clear and appropriate for a protocol paper.

Is the study design appropriate for the research question?

Yes

Is the rationale for, and objectives of, the study clearly described?

Yes

Are sufficient details of the methods provided to allow replication by others?

Yes

Are the datasets clearly presented in a useable and accessible format?

Yes

Reviewer Expertise:

Low calorie diet evaluation - however I'm not a health economist or statistician so could not provide a detailed review of these sections.

I confirm that I have read this submission and believe that I have an appropriate level of expertise to confirm that it is of an acceptable scientific standard.
